# Co-Designing Caregiver-Centered Care: Training the Health Workforce to Support Family Caregivers

**DOI:** 10.1093/geroni/igaa057.051

**Published:** 2020-12-16

**Authors:** Jasneet Parmar, Lisa Poole, Sharon Anderson, Pollard Cheryl, Wendy Duggleby, Lesley Charles, Suzette Brémault-Phillips, Jayna Holyroyd-Leduc

**Affiliations:** 1 University of Alberta, Edmonton, Alberta, Canada; 2 Dementia Connections Magazine, Calgary, Alberta, Canada; 3 McEwan University, Edmonton, Alberta, Canada; 4 University of Calgary, Calgary, Alberta, Canada

## Abstract

Family caregivers [FCGs] provide over 80% of the care for people with dementia, chronic illness and impairments. Despite evidence of their contributions and consequences of caregiving, support for FCGs has not been a health system priority. Our innovative solution, to reduce caregiver distress and support caregivers’ wellbeing, is to educate the health workforce in a meaningful manner based on evidence. We validated Caregiver-Centered Care Core Competencies required to address the gap between what FCGs report they need and preparation of healthcare providers to meet those needs. This competency-based education will prepare healthcare providers to effectively identify, engage, assess, and support FCGs, and address the inconsistent system of supports for FCGs. We co-designed our Caregiver Centered Care Education using effective practices for dementia education for health workforce and co-design. We engaged over 60 multi-level, interdisciplinary stakeholders with expertise in health workforce education, frontline healthcare, dementia care, health policy, and family caregiving. We ensured that we included FCGs of people living with dementia. The teaching/learning resources include competency-aligned educational modules, multimedia resources, and facilitators guide. As the hallmark of effective education is content relevant to learners’ needs and contexts, our education is designed flexibly, to be tailored to settings and learners. We are pilot testing the Caregiver-Centered Care Education, for acceptability and effectiveness, in five contexts: primary care, acute care, homecare, supportive living, and long-term care. Our education will support Caregiver-Centered Care in all settings providing dementia-related care. Health workforce support will be essential to maintain FCG wellbeing and sustain family caregiving.

